# Supplementary material for: Novel Driver Strength Index highlights important cancer genes in TCGA PanCanAtlas patients
Source: PeerJ. 2022 Aug 11;10:e13860. doi: 10.7717/peerj.13860 (PMC9375969; doi:10.7717/peerj.13860)
Supplement: Supplemental Information 2 [file peerj-10-13860-s002.zip › COHORTS/genes plots/detailed/2021_8_16_14_9_distribution_events_detailed_2.pdf]

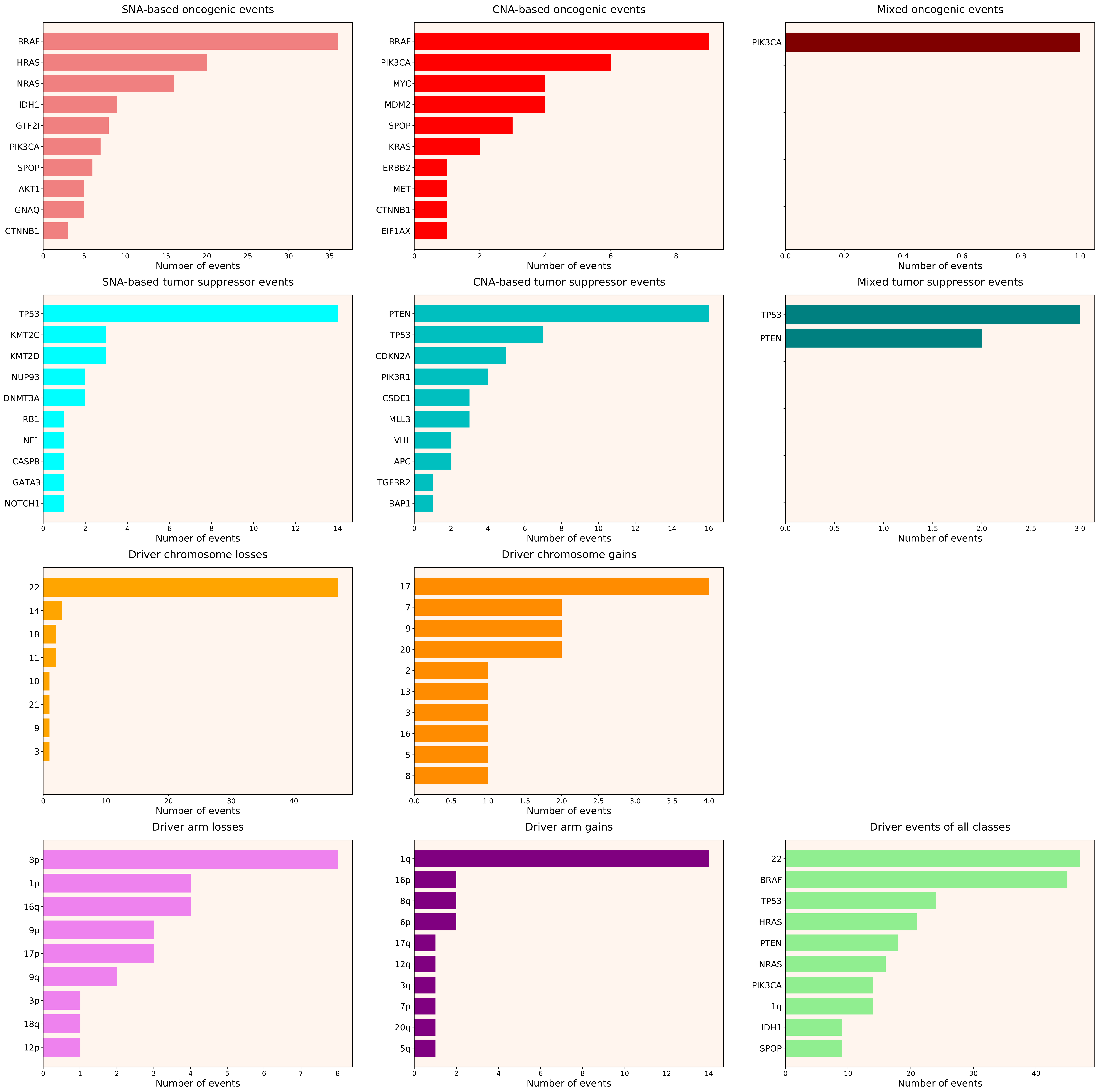
SNA-based oncogenic events
BRAF
HRAS
NRAS
IDH1
GTF2I
PIK3CA
SPOP
AKT1
GNAQ
CTNNB1
Number of events
CNA-based oncogenic events
BRAF
PIK3CA
MYC
MDM2
SPOP
KRAS
ERBB2
MET
CTNNB1
EIF1AX
Number of events
Mixed oncogenic events
PIK3CA
Number of events
SNA-based tumor suppressor events
TP53
KMT2C
KMT2D
NUP93
DNMT3A
RB1
NF1
CASP8
GATA3
NOTCH1
Number of events
CNA-based tumor suppressor events
PTEN
TP53
CDKN2A
PIK3R1
CSDE1
MLL3
VHL
APC
TGFBR2
BAP1
Number of events
Mixed tumor suppressor events
TP53
PTEN
Number of events
Driver chromosome losses
22
14
18
11
10
21
9
3
Number of events
Driver chromosome gains
17
7
9
20
2
13
3
16
5
8
Number of events
Driver arm losses
8p
1p
16q
9p
17p
9q
3p
18q
12p
Number of events
Driver arm gains
1q
16p
8q
6p
17q
12q
3q
7p
20q
5q
Number of events
Driver events of all classes
22
BRAF
TP53
HRAS
PTEN
NRAS
PIK3CA
1q
IDH1
SPOP
Number of events
